# Retention and losses of ultraviolet-sensitive visual pigments in bats

**DOI:** 10.1038/s41598-018-29646-6

**Published:** 2018-08-09

**Authors:** Longfei Li, Hai Chi, Haonan Liu, Yu Xia, David M. Irwin, Shuyi Zhang, Yang Liu

**Affiliations:** 1 Key Laboratory of Zoonosis of Liaoning Province, College of Animal Science and Veterinary Medicine, Shenyang Agricultural University, Shenyang, 110866 China; 2 College of Food Science, Shenyang Agricultural University, Shenyang, 110866 China; 3 Department of Laboratory Medicine and Pathobiology, University of Toronto, Toronto, ON M5S 1A8 Canada

## Abstract

Ultraviolet (UV)-sensitive visual pigment and its corresponding ability for UV vision was retained in early mammals from their common ancestry with sauropsids. Subsequently, UV-sensitive pigments, encoded by the short wavelength-sensitive 1 (*SWS1*) opsin gene, were converted to violet sensitivity or have lost function in multiple lineages during the diversification of mammals. However, many mammalian species, including most bats, are suggested to retain a UV-sensitive pigment. Notably, some cave-dwelling fruit bats and high duty cycle echolocating bats have lost their *SWS1* genes, which are proposed to be due to their roosting ecology and as a sensory trade-off between vision and echolocation, respectively. Here, we sequenced *SWS1* genes from ecologically diverse bats and found that this gene is also non-functional in both common vampire bat (*Desmodus rotundus*) and white-winged vampire bat (*Diaemus youngi*). Apart from species with pesudogenes, our evolutionary and functional studies demonstrate that the SWS1 pigment of bats are UV-sensitive and well-conserved since their common ancestor, suggesting an important role across major ecological types. Given the constrained function of SWS1 pigments in these bats, why some other species, such as vampire bats, have lost this gene is even more interesting and needs further investigation.

## Introduction

Ultraviolet (UV) light is perceived by diverse vertebrates to facilitate several behaviors, such as foraging^1,2^. The short wavelength-sensitive 1 (SWS1) visual pigment of ancestral vertebrates has been demonstrated to be UV-sensitive with a maximum absorption wavelength (λ_max_) of 361 nm^3^. UV sensitivity was then preserved in a variety of vertebrate taxa, with λ_max_ values ranging from 355 to 371 nm^4^. In early mammals, the SWS1 pigment inherited from their amniote ancestor retained UV sensitivity (λ_max_ = 359 nm)^4^. Subsequently, the *SWS1* gene was lost in the ancestor of monotremes, the only mammalian group that possesses a functional short wavelength-sensitive 2 (*SWS2*) opsin gene, after their split from therians (marsupials and placentals)^5^. In contrast, the common ancestor of therians lost *SWS2*, but kept the *SWS1* that encoded an UV-sensitive pigment^4,5^.

UV vision was initially thought to be rare in extant mammals, with only a few species, such as some bats, eulipotyphlans, rodents and marsupials, reported to have retained this sensitivity^6–10^. However, the recent finding, based on extensive genomic sequencing, suggests that UV-sensitive SWS1 pigments are more widely distributed in mammals than previously thought, with violet-sensitive pigments being derived multiple times^11^. Many mammals, however, have lost their *SWS1* genes^11,12^, and thus UV/violet sensitivity, and is probably associated with having a nocturnal lifestyle^12^. Intriguingly, nocturnal activity itself cannot fully explain the losses of *SWS1* as not all nocturnal species have lost this gene, suggesting other lineage-specific mechanisms potentially play roles^12^.

Bats (order Chiroptera) possess the second largest number of species, after rodents, within Mammals^13^ and are well-known for the special sensory adaptation, that is, advanced laryngeal echolocation^14^. Echolocation and ultrasonic hearing, processed using an enlarged auditory cortex in the brain, helps insectivorous bats detect and capture prey at night^15^. Old World fruit bats (family Pteropodidae), on the other hand, do not use laryngeal echolocation and rely largely on vision with enlarged eyes and visual cortex^15^. The pteropodid bats share a common ancestor with echolocating bats from superfamily Rhinolophoidea to form a suborder called Yinpterochiroptera^16^. Within the rhinolophoid species, the horseshoe and roundleaf bats (families Rhinolophidae and Hipposideridae) are insectivorous and use a sophisticated high duty cycle echolocation^17^. The other suborder of bats, Yangochiroptera, includes species (except *Pteronotus parnellii*) that use low duty cycle echolocation^16,17^, and have diverse diets, with many species from the family Phyllostomidae being frugivorous, nectarivorous, omnivorous and even sanguivorous^18^.

The majority of *SWS1* genes from both echolocating and non-echolocating bat species were predicted to be UV-sensitive^11,19–22^. However, the exact λ_max_ values (phenotypes) of bat SWS1 pigments have not been experimentally verified. Until now, 17 amino-acid sites have been identified as being critical for the spectral sensitivities of SWS1 pigments^4^. However, these sites have a limited ability to predict λ_max_ values, based on gene sequences, for mammalian SWS1 pigments^23^. Interestingly, some species from the suborder Yinpterochiroptera, were reported to have independently lost their *SWS1* genes^11,20,24^. Specifically, losses of *SWS1* in the horseshoe and roundleaf bats were suggested to be sensory trade-offs with their sophisticated high duty cycle echolocation^20^. Individual losses of *SWS1* genes were also noted in some Old World fruit bats, which were hypothesized to be related to their cave-roosting habit^20^. Here we sequenced the complete *SWS1* coding regions from several ecologically diverse bat species, expressed these opsins, and then determined the spectral sensitivities of the visual pigments to study the roles of bat SWS1 pigments and also provide a functional supplement to the mammalian SWS1 pigments.

## Results and Discussion

To investigate the spectral sensitivities of bat SWS1 pigments, we successfully amplified and sequenced complete *SWS1* coding regions *de novo* for three species of bats and completed the missing 5′ and 3′ portions of sequences for an additional five species. Sequences from three (*Cynopterus sphinx*, *Megaderma spasma* and *Rhinopoma hardwickii*) of the eight species come from three different families of the suborder Yinpterochiroptera, while the other five species (*Chaerephon plicatus*, *Miniopterus fuliginosus*, *Pipistrellus abramus*, *Leptonycteris yerbabuenae* and *Artibeus lituratus*) are from four families of the suborder Yangochiroptera. Moreover, these eight species are ecologically diversified, representing non-echolocating Old World fruit bats (*C*. *sphinx*), echolocating New World fruit bats (*L*. *yerbabuenae* and *A*. *lituratus*) and insectivorous bats that use low duty cycle echolocation (*M*. *spasma* and *R*. *hardwickii*, *C*. *plicatus*, *M*. *fuliginosus* and *P*. *abramus*) (Supplementary Table 1). No frame-shift indels or premature stop codons were observed in the coding sequences from these eight species, suggesting that they have functional SWS1 pigments (Supplementary Fig. 1). Acquisition of the full-length coding sequences from these diverse bats enabled the subsequent evolutionary analyses and functional assays of the visual pigments.

**Figure 1:**
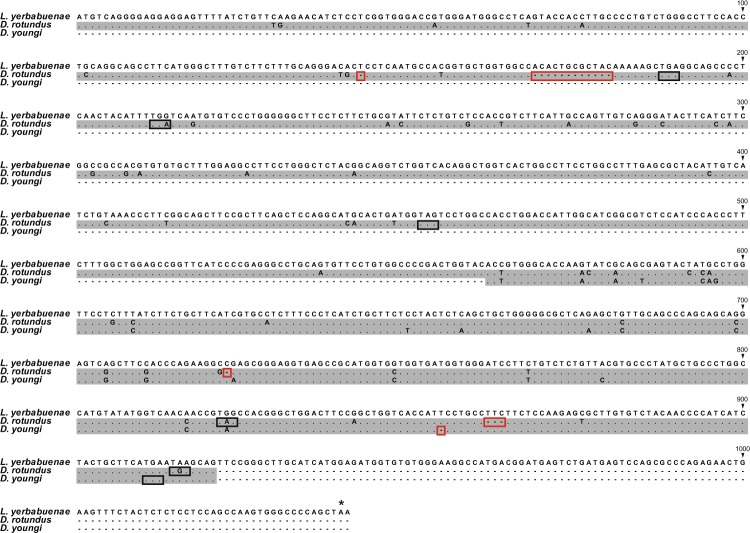
Alignment of vampire bat *SWS1* sequences. The nectar-feeding bat *Leptonycteris yerbabuenae* is a close relative of vampire bats and has an intact *SWS1* coding region (“*” indicates the stop codon). Numbering of the alignment is according to the full-length *L. yerbabuenae* sequence. Only partial coding regions were sequenced from the two vampire bats, and are shown with a gray background. Deletion events in the vampire bat sequences are indicated by the red boxes and premature stop codons by the black boxes.

In contrast to the above eight species, only partial coding regions could be amplified from two vampire bats, *Desmodus rotundus* and *Diaemus youngi* (Phyllostomidae) (Supplementary Table 1). Surprisingly, both of these sequences contained one or more deletions that lead to frame-shifts. Specifically, two of the four deletion events in the common vampire bat (*Desmodus rotundus*) result in frame-shifts, and there are a total of five premature stop codons in this gene. In the white-winged vampire bat (*Diaemus youngi*), there is one deletion, which is different from those in the common vampire bat, which causes a frame-shift that leads to a premature stop codon (Fig. 1). These results demonstrate that the two vampire bats independently lost their *SWS1* genes after speciation. Previously, some Old World fruit bats from the family Pteropodidae and all high duty cycle echolocating bats from the families Rhinolophidae and Hipposideridae have been reported to have lost their UV pigment gene^20^. A sensory trade-off hypothesis, between vision and echolocation, was proposed for rhinolophid and hipposiderid bats that utilize specialized high duty cycle echolocation to explain the loss of the *SWS1* gene in these species^20^.

To examine selective pressure (ratio of non-synonymous and synonymous substitution rates, or *ω*) acting on bat SWS1 pigments, we first carried out an evolutionary analysis of the *SWS1* sequences (no pseudogenes included) from both bats and other mammals (Supplementary Fig. 2A). Results from the clade model C analysis using PAML 4.9 show a subset of sites that are under different selective pressure in bats (*ω* = 0.21) compared to other mammalian species (0.32) (*P* < 0.001). Notably, even sites in *SWS1* that are under purifying selection in both groups are more strongly conserved in bats. Additional comparisons between bats and species from single orders (i.e., Carnivora, Perissodactyla, Cetartiodactyla, Eulipotyphla, Primates and Rodentia) retain support the conclusion that the *SWS1* genes in bats experienced stronger purifying selection than in other mammals, with the exception of eulipotyphlans (Table 1). A sliding window analysis was then conducted to calculate the *ω* values for species from each order. Results of this analysis show that the estimated *ω* values are higher in species from the orders Carnivora, Perissodactyla, Cetartiodactyla and Primates than in bats, while no obvious difference among bats, eulipotyphlans and rodents (Supplementary Fig. 2B), which is a pattern similar to that derived from clade model C. Previous findings, based on other evolutionary models in PAML, have shown purifying selection on bat *SWS1* genes^20,22^. The results of our study further reveal that the negative selection acting on bat *SWS1* is even stronger than on other mammals (except eulipotyphlan species), which suggests that the spectral sensitivities of SWS1 pigments from these bats have probably not shifted much.

**Table 1:** Selective pressures on *SWS1* genes from bats and other mammalian groups.

Comparison	Model	Log-likelihood	Parameter	*P* value
Bats vs other mammals	Clade model C	−14832.56	p_0_ = 0.64, p_1_ = 0.08, p_2_ = 0.29 *ω*_0_ = 0.02, *ω*_1_ = 1, ***ω***_**2**_ = **0.32**, ***ω***_**3**_ = **0.21**	<0.001
	M1a	−14997.84	p_0_ = 0.8, p_1_ = 0.2 *ω*_0_ = 0.06, *ω*_1_ = 1	
Bats vs Carnivora	Clade model C	−4054.77	p_0_ = 0.62, p_1_ = 0.06, p_2_ = 0.32 *ω*_0_ = 0, *ω*_1_ = 1, ***ω***_**2** = _**0.27**, ***ω***_**3** = _**0.18**	<0.001
	M1a	−4066.4	p_0_ = 0.89, p_1_ = 0.11 *ω*_0_ = 0.05, *ω*_1_ = 1	
Bats vs Perissodactyla	Clade model C	−3461.67	p_0_ = 0.78, p_1_ = 0.13, p_2_ = 0.09 *ω*_0_ = 0.03, *ω*_1_ = 1, ***ω***_**2** = _**4.56**, ***ω***_**3** = _**0**	<0.001
	M1a	−3483.02	p_0_ = 0.88, p_1_ = 0.12 *ω*_0_ = 0.05, *ω*_1_ = 1	
Bats vs Cetartiodactyla	Clade model C	−4350.09	p_0_ = 0.86, p_1_ = 0.11, p_2_ = 0.03 *ω*_0_ = 0.04, *ω*_1_ = 1, ***ω***_**2** = _**3.3**, ***ω***_**3** = _**0.01**	<0.001
	M1a	−4364.07	p_0_ = 0.86, p_1_ = 0.14 *ω*_0_ = 0.04, *ω*_1_ = 1	
Bats vs Eulipotyphla	Clade model C	−3431.44	p_0_ = 0.81, p_1_ = 0, p_2_ = 0.19 *ω*_0_ = 0.01, *ω*_1_ = 1, ***ω***_**2** = _**0.18**, ***ω***_**3** = _**0.52**	<0.001
	M1a	−3447.71	p_0_ = 0.89, p_1_ = 0.11 *ω*_0_ = 0.03, *ω*_1_ = 1	
Bats vs Primates	Clade model C	−5630.67	p_0_ = 0.71, p_1_ = 0.1, p_2_ = 0.19 *ω*_0_ = 0.03, *ω*_1_ = 1, ***ω***_**2** = _**0.85**, ***ω***_**3** = _**0.09**	<0.001
	M1a	−5660.64	p_0_ = 0.82, p_1_ = 0.18 *ω*_0_ = 0.06, *ω*_1_ = 1	
Bats vs Rodentia	Clade model C	−6499.92	p_0_ = 0.66, p_1_ = 0.05, p_2_ = 0.28 *ω*_0_ = 0.01, *ω*_1_ = 1, ***ω***_**2** = _**0.34**, ***ω***_**3** = _**0.23**	<0.001
	M1a	−6539.31	p_0_ = 0.85, p_1_ = 0.15 *ω*_0_ = 0.06, *ω*_1_ = 1	

The estimated *ω*_2_ and *ω*_3_ values for background and foreground clades are shown in bold.

**Figure 2:**
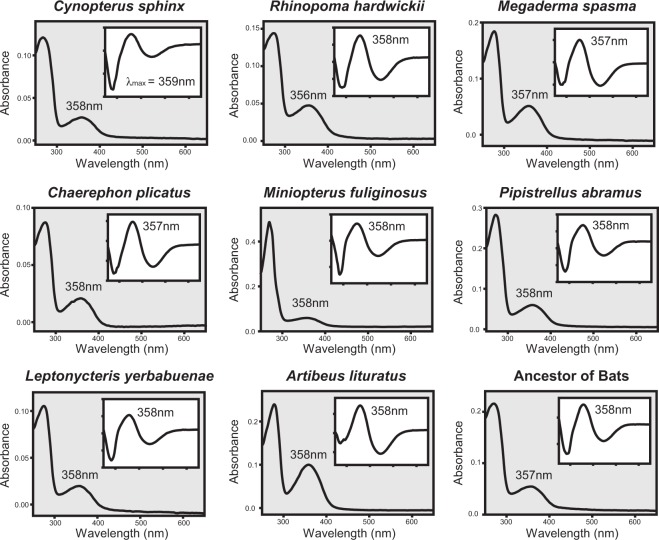
Spectral tuning of bat SWS1 pigments. Spectral sensitivities, with λ_max_ values indicated, for SWS1 pigments from eight extant bat species and the resurrected bat ancestor are shown. The inset exhibits the dark minus acid difference spectrum for each pigment.

**Figure 3:**
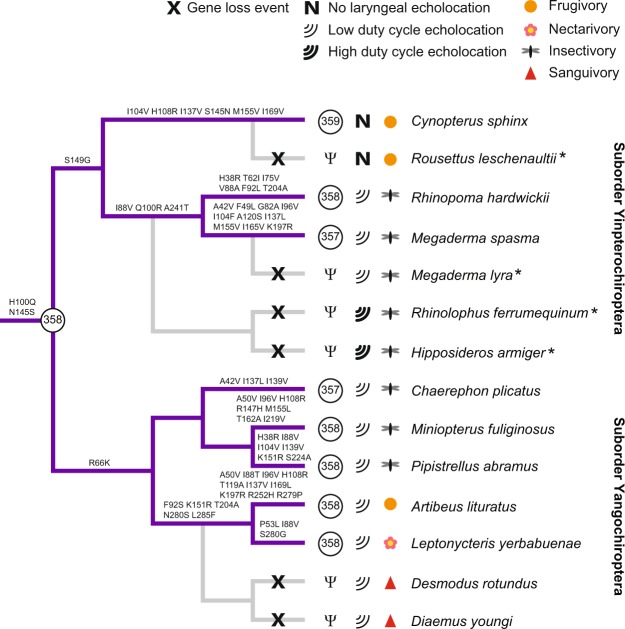
Evolution of UV pigments in bats. λ_max_ values of the UV pigments, type of echolocation, and diet are mapped onto the chiropteran phylogenetic tree. Absence of *SWS1* (pseudogene) is indicate by Ψ. Amino-acid substitutions in the SWS1 opsins are indicated on the lineages, with only sites between transmembrane I and VII shown^44^. Numbering of the sites follows that of bovine rhodopsin. Losses of *SWS1* genes in bats are indicated by gray branches in the tree. Gene loss events which have been published in previous studies are denoted by “*” after the species name^11,20^.

We used *in vitro* assays on purified recombinant SWS1 photopigments to determine their spectral sensitivities. Our results demonstrate that SWS1 from all eight bat species examined are UV-sensitive with λ_max_ values ranging from 357 to 359 nm (Fig. 2). Moreover, the resurrected SWS1 pigment of the bat ancestor is also UV-sensitive (λ_max_ = 358 nm), demonstrating a conserved function in bats with only minor λ_max_-shifts (Figs 2 and 3). Bat SWS1, therefore, is a new group of UV-sensitive pigments in mammals that has been functionally verified by *in vitro* assay, in addition to the previously characterized pigments from rodents (*Mus musculus* and *Rattus norvegicus*) and marsupials (*Didelphis aurita* and *Sminthopsis crassicaudata*), which have λ_max_ values of 358–359 nm and 362–363 nm respectively^25–28^. All of the bat SWS1 pigments studied here are UV-sensitive, with differences of only 2 nm or less in spectral sensitivity, and are from species spanning various ecological types, including a non-echolocating fruit bat and low duty cycle echolocating bats feeding on fruits, nectar or insects (Fig. 3). The conserved UV pigments from the major clades of order Chiroptera examined in this study add further support to the hypothesis that UV sensitivity mediated by SWS1 pigment is advantageous and important for specific bats^7,22^.

In contrast to the functional conservation of UV pigments in these species, some other bats have independently lost their *SWS1* genes^11,20,24^. Notably, vampire bats, the only group of sanguivorous mammals^29^, have lost their *SWS1* genes while other closely related phyllostomid species, such as *L. yerbabuenae* (λ_max_ = 358 nm) and *A. lituratus* (λ_max_ = 358 nm), retain functional copies. UV sensitivity through SWS1 pigment has been suggested to be useful for several activities such as detection of insects and flowers and for orientation^7,20–22^. While the reason underlying these losses of UV-sensitive pigments in vampire bats is not clear, it is possibly related to their changed foraging targets, which are mammals and birds^30,31^. In addition, the *SWS1* gene from the Indian false vampire bat (*Megaderma lyra*) has also been reported to be a pseudogene, based on a low-coverage genome sequence^11,32^. We re-sequenced part of the *SWS1* coding region from this species and confirmed that it is a pseudogene (Supplementary Table 1). However, a congener species, the lesser false vampire bat (*Megaderma spasma*), retains a functional UV pigment (λ_max_ = 357 nm), with negligible functional difference from the bat ancestor (Fig. 3). Therefore the reasons for the independent losses of *SWS1* genes in the vampire bats and also in the Indian false vampire bat are likely specifically related to their individual ecologies, and further studies are necessary to elucidate the causes.

## Methods

### Bat *SWS1* sequencing

To examine the phenotypes of SWS1 pigments in bats of the order Chiroptera, complete coding sequences from representative species are required. Here we studied 11 species from the suborders Yinpterochiroptera, *Cynopterus sphinx* (family Pteropodidae), *Rhinopoma hardwickii* (Rhinopomatidae), *Megamerda spasma* and *M. lyra* (Megadermatidae), and Yangochiroptera, *Chaerephon plicatus* (Molossidae), *Miniopterus fuliginosus* (Miniopteridae), *Pipistrellus abramus* (Vespertilionidae), and four additional species, *Leptonycteris yerbabuenae*, *Artibeus lituratus*, *Desmodus rotundus* and *Diaemus youngi* from the family Phyllostomidae. Bat sample collection (wing membrane and eye tissues) for this study was approved by the Animal Ethics Committee of Shenyang Agricultural University, and the experiments were performed following the guidelines and regulations.

Eye tissue from *C. sphinx* was used for amplification of the *SWS1* coding region. Total RNA extraction and first-strand cDNA synthesis were conducted using RNAiso Plus and PrimeScript II reagents (TaKaRa). Wing membrane tissues from the other ten species were collected for amplification of *SWS1* exons, separately, from genomic DNA. The TIANamp Kit (Tiagen) was used for genomic DNA extraction. Eye cDNA from *R. hardwickii* was also synthesized and used as a PCR template, since specific amplification of *SWS1* exons failed using genomic DNA for this species. Detailed information on primer pairs and corresponding annealing temperatures are provided in Supplementary Tables 1 and 2. PCR amplicons were purified, ligated into pGEM-T Easy cloning vector (Promega), transformed using DH5α competent cells (Tiagen), and then sequenced by an ABI 3730 machine (Applied Biosystems).

### Evolutionary analyses on *SWS1* genes

For the evolutionary analyses of the mammalian *SWS1* genes, 73 additional complete coding sequences were obtained from NCBI (www.ncbi.nlm.nih.gov) and Ensembl (www.ensembl.org). These sequences represent species from 14 orders of mammals and enable comparisons of the selective pressures between bats and other groups (Supplementary Table 3).

A total of 81 mammalian sequences (no pseudogenes included) were used to determine whether the selective pressures acting on *SWS1* genes were different in bats compared to other species. Sequences were aligned using Clustal W in MEGA 5 software^33^ and then checked by eye. Clade model C was implemented using CODEML in PAML 4.9^34,35^. This model allows a site class with different *ω* values between the focal clade (*ω*_3_ assigned to the foreground) and the others (*ω*_2_ to the background). In contrast, the null model, called M1a, assumes nearly neutral evolution for this gene in all species. The superior model (clade model C or M1a) for the data was identified using a likelihood ratio test. The species tree for these 81 mammals used in this analysis was based on published phylogenies^18,36–41^. Clade model C was also applied to compare *ω* values between bats and species from each of the orders Carnivora, Perissodactyla, Cetartiodactyla, Eulipotyphla, Primates or Rodentia, separately.

To visualize *ω* values across the *SWS1* gene sequence, a sliding window analysis was conducted using the software SWAAP 1^42^. By using a window size of 90 bp and a step size of 18 bp, *ω* values for the *SWS1* genes within each mammalian order (Chiroptera, Carnivora, Perissodactyla, Cetartiodactyla, Eulipotyphla, Primates or Rodentia) were calculated, respectively.

### *In vitro* assays for bat SWS1 pigments

The 81 species dataset was used to infer the ancestral SWS1 sequence for bats. The best-fitting amino-acid model for the data is JTT + I + Γ, which was selected by ProtTest 3 software according to the corrected Akaike information criterion^43^. The ancestral sequence for bat SWS1 was reconstructed under the best-fitting model and the species topology by using CODEML^34^.

Complete coding sequences of eight bat *SWS1* genes and the inferred sequence of the common ancestor of bats were synthesized and ligated into the pcDNA3.1 expression vector (Invitrogen) using the *Hin*dIII and *Xho*I restriction sites. A Kozak sequence “CCACC” was added in front of the start codon for each gene. A short sequence “ACA GAG ACC AGC CAA GTG GCG CCT GCC”, encoding “TETSQVAPA”, was added prior to the stop codon, and used as an epitope tag for protein purification. Plasmids containing the *SWS1* genes were transfected into HEK293T cells individually using Xfect reagent (Clontech). Expressed SWS1 opsins were harvested from cells 48 h after transfection. Visual pigments were regenerated by incubating the cells together with 11-*cis*-retinal (Storm Eye Institute, Medical University of South Carolina). Visual pigments were then solubilized using n-Dodecyl β-D-maltoside and purified with the Rho 1D4 monoclonal antibody (University of British Columbia) following published procedures^26^. Absorbance spectra of the SWS1 pigments were measured by a spectrophotometer (U-3900, Hitachi) in the dark before and after sulfuric acid denaturation orderly^26^.

## Electronic supplementary material

Supplementary information
