# Supplementary material for: Retention and losses of ultraviolet-sensitive visual pigments in bats
Source: Sci Rep. 2018 Aug 9;8:11933. doi: 10.1038/s41598-018-29646-6 (PMC6085362; doi:10.1038/s41598-018-29646-6)
Supplement: Supplementary file 1 — Supplementary information [file 41598_2018_29646_MOESM1_ESM.pdf]

Supplementary Information

Retention and losses of ultraviolet-sensitive visual pigments in bats

Longfei Li, Hai Chi, Haonan Liu, Yu Xia, David M. Irwin, Shuyi Zhang & Yang Liu

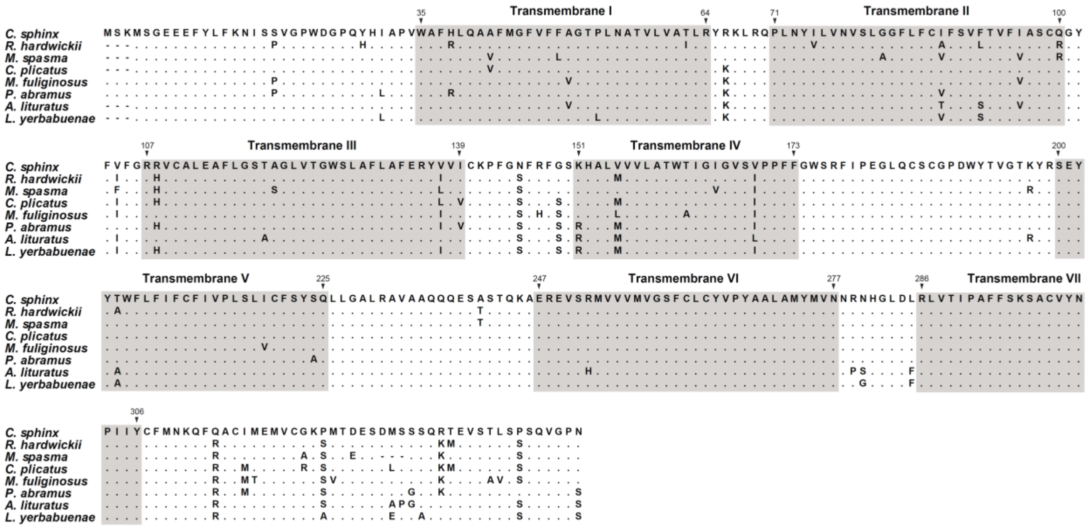

Supplementary Figure 1. Alignment of complete SWS1 opsins from bats used in this study. Transmembrane domains I to VII of the bat SWS1 sequences are predicted according to the corresponding positions in bovine rhodopsin[1].

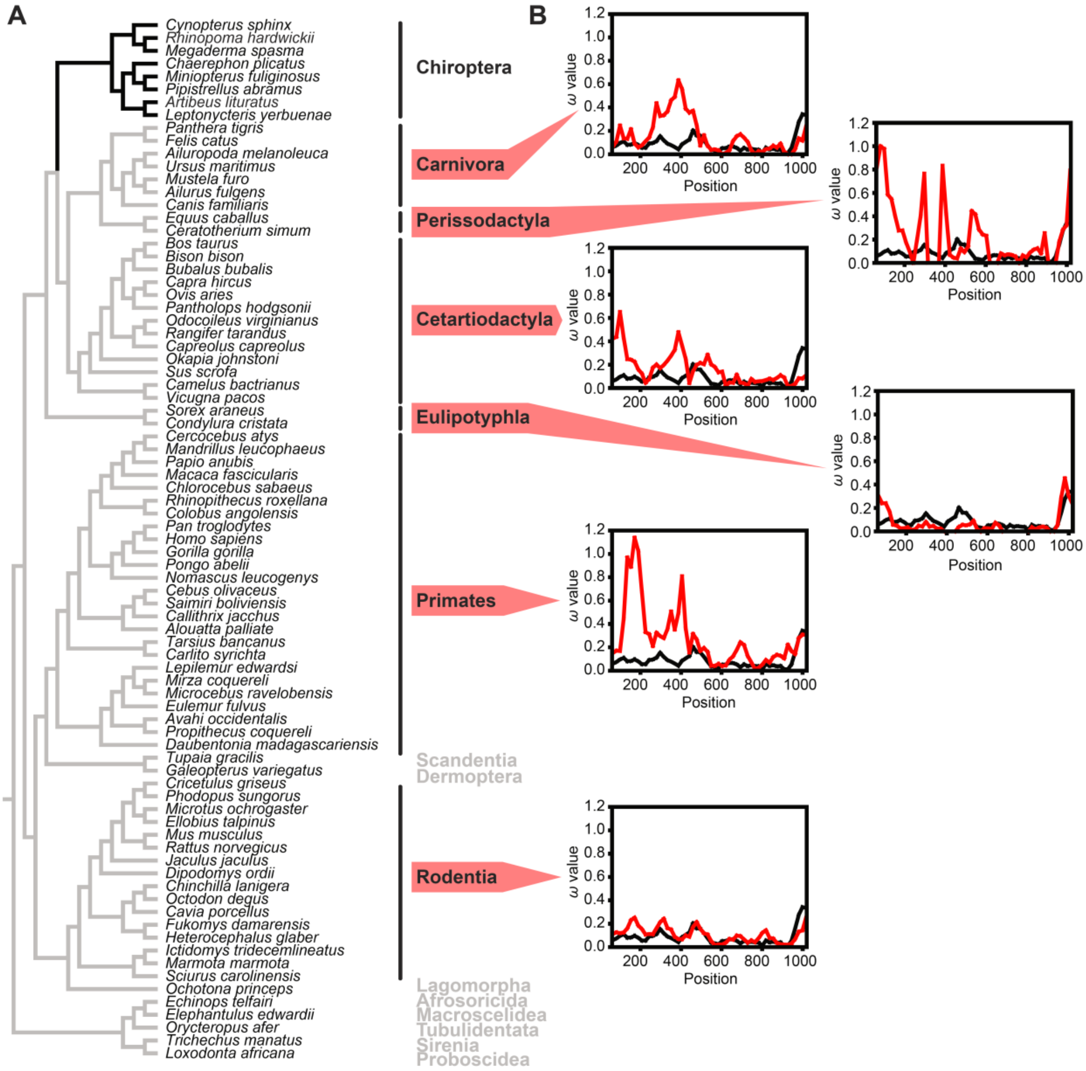

Supplementary Figure 2. Mammalian *SWS1* genes used in the molecular evolutionary analyses. (A) Phylogeny of mammalian species used in the clade model C analysis. The focal clade of bats is indicated in black with other mammals are in gray. (B) Comparisons of the ω values between bats (black line) and other groups (red line) are shown.

Family	Bat species	Echolocation	Diet	Exon 1	Exon 1-3	Exon 3-4	Exon 4	Exon 5	Accession No.
Pteropodidae	*Cynopterus sphinx*	No laryngeal echolocation[2]	Frugivory[3]	BatS1-F1<br>BatS1-R1<br>(55 ℃)					MG904970
Pteropodidae	*Rousettus leschenaultii*	No laryngeal echolocation[4]	Frugivory[3]		EU912381[‡]				
Rhinolophidae	*Rhinolophus ferrumequinum*	High duty cycle echolocation[5]	Insectivory[6]		EU912380[‡]				
Hipposideridae	*Hipposideros armiger*	High duty cycle echolocation[7]	Insectivory[8]		EU912368[‡]				
Rhinopomatidae	*Rhinopoma hardwickii*	Low duty cycle echolocation[9]	Insectivory[10]	BatS1-1-F1<br>BatS1-1-R1<br>(55 ℃)	RhaS1-1-4-F1<br>SINRb[†]<br>(55 ℃)		BatS1-4-5-F1<br>BatS1-4-5-R1<br>(55 ℃)		MG904968<br>MG904965
Megadermatidae	*Megaderma spasma*	Low duty cycle echolocation[11]	Insectivory[12]	BatS1-1-F2<br>MspS1-1-R1<br>(55 ℃)<br>BatS1-1-F3[*]<br>MspS1-1-R2[*]<br>(55 ℃)	EU912378[‡]		BatS1-4-5-F1<br>BatS1-4-5-R1<br>(58.8 ℃)		MG904967
Megadermatidae	*Megaderma lyra*	Low duty cycle echolocation[11]	Insectivory[13]			SDC2F[†]<br>SINRb[†]<br>(56 ℃)			MH469713
Phyllostomidae	*Desmodus rotundus*	Low duty cycle echolocation[14]	Sanguivory[15]	BatS1-1-F1<br>BatS1-1-R1<br>(55 ℃)	DroS1-1-3-F1<br>DroS1-1-3-R1<br>(58.5 ℃)	SDC2F[†]<br>SINRb[†]<br>(56 ℃)	BatS1-4-5-F1<br>BatS1-4-R1<br>(52 ℃)		MG904975
Phyllostomidae	*Diaemus youngi*	Low duty cycle echolocation[14]	Sanguivory[16]			SDC2F[†]<br>BatS1-3-4-R1<br>(55 ℃)			MG904974
Phyllostomidae	*Leptonycteris yerbabuenae*	Low duty cycle	Nectarivory[18]	BatS1-1-F1<br>BatS1-1-R1	LyeS1-1-3-F1<br>LyeS1-1-3-R1	SDC2F[†]<br>SINRb[†]	BatS1-4-F1<br>BatS1-4-R1	BatS1-5-F1	MG904971

		echolocation[17]		(55 ℃)	(55 ℃)<br>LyeS1-1-3-F2[*]<br>SDC1R[*†]<br>(55 ℃)	(56 ℃)	(52 ℃)	BatS1-5-R1<br>(52 ℃)	
Phyllostomidae	*Artibeus lituratus*	Low duty cycle echolocation[19]	Frugivory[20]	BatS1-1-F1<br>BatS1-1-R1<br>(55 ℃)	EU912372[‡]		BatS1-4-5-F2<br>BatS1-4-5-R2<br>(55 ℃)<br>BatS1-4-5-F1[*]<br>BatS1-4-5-R3[*]<br>(55 ℃)		MG904972
Molossidae	*Chaerephon plicatus*	Low duty cycle echolocation[21]	Insectivory[22]	BatS1-1-F1<br>BatS1-1-R1<br>(55 ℃)	EU912359[‡]		BatS1-4-F1<br>BatS1-4-R1<br>(52.5 ℃)	BatS1-4-F1<br>BatS1-4-5-R1<br>(58.6 ℃)<br>BatS1-5-F2[*]<br>BatS1-5-R2[*]<br>(55 ℃)	MG904973
Miniopteridae	*Miniopterus fuliginosus*	Low duty cycle echolocation[23]	Insectivory[6]	MfuS1-1-F1<br>MfuS1-1-R1<br>(55 ℃)	EU912376[‡]		BatS1-4-F1<br>BatS1-4-R1<br>(56 ℃)	BatS1-5-F1<br>BatS1-5-R1<br>(53.5 ℃)	MG904969
Vespertilionidae	*Pipistrellus abramus*	Low duty cycle echolocation[24]	Insectivory[25]	BatS1-1-F1<br>BatS1-1-R1<br>(55 ℃)	EU912360[‡]		BatS1-4-F2<br>BatS1-3-4-R1<br>(55 ℃)	BatS1-5-F1<br>BatS1-4-5-R1<br>(53.5 ℃)	MG904966

Supplementary Table 1. Bat species and ecologies, PCR primer pairs used and GenBank accession numbers for *SWS1*. The symbol "*" indicates primers used for nested PCR. PCR primers and bat *SWS1* sequences published in previous literature are marked by "†" and "‡" respectively[26]. Annealing temperatures for the PCR reactions are shown in parentheses.

Primer name	Primer sequence		
BatS1-F1	5'	CTTAGGAAGCTTAGGATCAGCACTG	3'
BatS1-R1	5'	GCTGGAACAGTAGCTTTATAGG	3'
BatS1-1-F1	5'	GACCATTCAGGGAACCCAAAGTG	3'
BatS1-1-R1	5'	GGCRAYRAAGACRGTGRAGACAGAG	3'
BatS1-1-F2	5'	GAYCATTCAGGKARCCCAAAAGTG	3'
MspS1-1-R1	5'	CACAAAGACGGTGAAGACAGAGAAG	3'
BatS1-1-F3	5'	AGTGGCTTTGGAGGMKGGGTTC	3'
MspS1-1-R2	5'	ACAGAACAGGAAGCCCGCCAG	3'
MfuS1-1-F1	5'	GGGAGGGTAACCTATAAGAGGAATC	3'
MfuS1-1-R1	5'	CTGCTCCCTGGTTCCAATCCTG	3'
RhaS1-1-4-F1	5'	CTCAACTACGTCCTGGTCAATGTGT	3'
DroS1-1-3-F1	5'	GGGAGGAGGAGTTTTATCTGTTTGA	3'
DroS1-1-3-R1	5'	ATTCCATGTCATCATTCTGCCTATC	3'
LyeS1-1-3-F1	5'	GGGGAGGAGGAGTTTTATCTGTTCA	3'
LyeS1-1-3-R1	5'	GCAGGGATGACTCTCTACCTTACTC	3'
LyeS1-1-3-F2	5'	GATGGGCCTCAGTACCACCTTG	3'
BatS1-3-4-R1	5'	TGTTYYYKYGGCTTCCCTTGTTC	3'
BatS1-4-5-F1	5'	GRGTCMTTYTGTCTCTGTTAYGTGCC	3'
BatS1-4-5-R1	5'	TCATCACGCCCCTTCCCATCTGTT	3'
BatS1-4-R1	5'	AAYTGGRAGGAYTAGAATGTARGGG	3'
BatS1-4-F1	5'	ARCCACGGGCTRGACYTRCGGC	3'
BatS1-4-5-F2	5'	ATGGTGGTGGTSATGGTGGG	3'
BatS1-4-5-R2	5'	CCAGCTGGAACGGTAGCTTTGTAGG	3'
BatS1-4-5-R3	5'	GCTGYAAAKAGTCCAATATGGC	3'
BatS1-4-F2	5'	CACCATTCCTGCCTTCTTCTCC	3'
BatS1-5-F1	5'	TGTYATCTCCTYTGYWCYGGTCTGC	3'
BatS1-5-R1	5'	GCTGGAACGGTAGCTTTGTAGG	3'
BatS1-5-F2	5'	GCTGCTTYTSACRRTGCTCC	3'
BatS1-5-R2	5'	ATAGTCCAATATGGCGCCTTA	3'
SFe*	5'	ATGTCAGRGGARGAGTTTTATCTGTTCAAG	3'
SDC1R*	5'	TATAGKACTCGCTGCGRTAYTTGGTGCC	3'
SDC2F*	5'	GCAGTGTTCCTGTGGYCCYGACTGGTAC	3'
SINRb*	5'	GGAACTGCTTATTCATGAAGCAGTAGATGATGGG	3'

Supplementary Table 2. Primer sequences used in this study. The "*" symbol indicates primers from the published literature[26].

Order	Species name	Accession No.
Carnivora	*Ailuropoda melanoleuca*	XM_002913423.3
Carnivora	*Canis familiaris*	XM_539386.4
Carnivora	*Felis catus*	XM_003983019.4
Carnivora	*Mustela furo*	XM_004741814.2
Carnivora	*Panthera tigris*	XM_007078061.2
Carnivora	*Ursus maritimus*	XM_008705847.1
Carnivora	*Ailurus fulgens*	LNAC01000049.1
Perissodactyla	*Ceratotherium simum*	XM_004418710.1
Perissodactyla	*Equus caballus*	XM_001502735.4
Cetartiodactyla	*Bison bison*	XM_010831706.1
Cetartiodactyla	*Bos taurus*	U92557.1
Cetartiodactyla	*Bubalus bubalis*	XM_006074392.1
Cetartiodactyla	*Camelus bactrianus*	XM_010947561.1
Cetartiodactyla	*Capra hircus*	XM_005679445.2
Cetartiodactyla	*Capreolus capreolus*	CCMK010160076.1
Cetartiodactyla	*Odocoileus virginianus*	XM_020914876.1
Cetartiodactyla	*Okapia johnstoni*	LVCL010093858
Cetartiodactyla	*Vicugna pacos*	XM_006202271.1
Cetartiodactyla	*Ovis aries*	XM_004008047.3
Cetartiodactyla	*Sus scrofa*	NM_214090.1
Cetartiodactyla	*Pantholops hodgsonii*	XM_005981411.1
Cetartiodactyla	*Rangifer tarandus*	FN808318
Eulipotyphla	*Condylura cristata*	XM_004677016.2
Eulipotyphla	*Sorex araneus*	XM_004608279.2
Primates	*Callithrix jacchus*	XM_002752031.3
Primates	*Carlito syrichta*	XM_008063590.1
Primates	*Cercocebus atys*	XM_012087993.1
Primates	*Chlorocebus sabaeus*	XM_007982851.1
Primates	*Colobus angolensis*	XM_011939561.1
Primates	*Gorilla gorilla*	XM_004046176.2
Primates	*Homo sapiens*	AH003620.2
Primates	*Macaca fascicularis*	AF158977.1
Primates	*Mandrillus leucophaeus*	XM_011995905.1
Primates	*Nomascus leucogenys*	XM_003261297.2
Primates	*Pan troglodytes*	NM_001009127.1
Primates	*Papio anubis*	XM_003896561.3
Primates	*Pongo abelii*	XM_002818421.2
Primates	*Propithecus coquereli*	XM_012664772.1
Primates	*Rhinopithecus roxellana*	XM_010378740.1

Primates	*Saimiri boliviensis*	U53875.1
Primates	*Avahi occidentalis*	JX867506.1
Primates	*Lepilemur edwardsi*	JX867476
Primates	*Microcebus ravelobensis*	JX867530
Primates	*Cebus olivaceus*	AH005810
Primates	*Alouatta palliate*	AH005790
Primates	*Daubentonia madagascariensis*	EF667282
Primates	*Eulemur fulvus*	AB111464
Primates	*Tarsius bancanus*	AB111463
Primates	*Mirza coquereli*	DQ191903-DQ191907
Scandentia	*Tupaia gracilis*	KU255121
Dermoptera	*Galeopterus variegatus*	XM_008585390.1
Rodentia	*Cavia porcellus*	AY552608.1
Rodentia	*Chinchilla lanigera*	XM_005402383.2
Rodentia	*Cricetulus griseus*	XM_003497518.3
Rodentia	*Dipodomys ordii*	XM_013019960.1
Rodentia	*Ellobius talpinus*	LOJH01047118
Rodentia	*Fukomys damarensis*	XM_010633453.1
Rodentia	*Heterocephalus glaber*	XM_004856389.1
Rodentia	*Ictidomys tridecemlineatus*	XM_005319430.1
Rodentia	*Jaculus jaculus*	XM_004661221.2
Rodentia	*Marmota marmota*	XM_015488317.1
Rodentia	*Microtus ochrogaster*	XM_005365745.1
Rodentia	*Mus musculus*	U49720.1
Rodentia	*Octodon degus*	XM_004642726.1
Rodentia	*Phodopus sungorus*	MCBN011439082
Rodentia	*Sciurus carolinensis*	DQ302163
Rodentia	*Rattus norvegicus*	Ensembl BLAT
Lagomorpha	*Ochotona princeps*	XM_004592516.1
Afrosoricida	*Echinops telfairi*	XM_004707803.1
Macroscelidea	*Elephantulus edwardii*	XM_006896064.1
Sirenia	*Trichechus manatus*	XM_004382643.2
Tubulidentata	*Orycteropus afer*	XM_007955413.1
Proboscidea	*Loxodonta africana*	AY686753.1

Supplementary Table 3. *SWS1* genes from other mammalian species.

References

1. Palczewski, K. *et al.* Crystal structure of rhodopsin: A G protein-coupled receptor. *Science* **289**, 739-45 (2000).
2. Zhang, W. *et al.* Role of olfaction in the foraging behavior and trial-and-error learning in short-nosed fruit bat, *Cynopterus sphinx*. *Behav Processes* **103**, 23-7 (2014).
3. Tang, Z., Sheng, L., Cao, M., Liang, B. & Zhang, S. Diet of *Cynopterus sphinx* and *Rousettus leschenaulti* in Xishuangbanna. *Acta Theriol Sinica* **25**, 367-372 (2005).
4. Raghurm, H., Thangadurai, C., Gopukumar, N., Nathar, K. & Sripathi, K. The role of olfaction and vision in the foraging behaviour of an echolocating megachiropteran fruit bat, *Rousettus leschenaulti* (Pteropodidae). *Mamm Biol* **74**, 9-14 (2009).
5. Jones, G. & Rayner, J.M.V. Foraging behavior and echolocation of wild horseshoe bats *Rhinolophus ferrumequinum* and *R. hipposideros* (Chiroptera, Rhinolophidae). *Behav Ecol Sociobiol* **25**, 183-191 (1989).
6. Funakoshi, K. & Takeda, Y. Food habits of sympatric insectivorous bats in southern Kyushu, Japan. *Mamm Stud* **23**, 49-62 (1998).
7. Fu, Z.Y., Tang, J., Jen, P.H. & Chen, Q.C. The auditory response properties of single-on and double-on responders in the inferior colliculus of the leaf-nosed bat, *Hipposideros armiger*. *Brain Res* **1306**, 39-52 (2010).
8. Zubaid, A. Food habits of *Hipposideros armiger* (Chiroptera: Rhinolophidae) from Peninsular Malaysia. *Mammalia* **52**, 585-588 (1988).
9. Simmons, J.A., Kick, S.A. & Lawrence, B.D. Echolocation and hearing in the mouse-tailed bat, *Rhinopoma hardwickei*: acoustic evolution of echolocation in bats. *J Comp Physiol A* **154**, 347-356 (1984).
10. Whitaker, J.O. & Yom-Tov, Y. The diet of some insectivorous bats from northern Israel. *Mamm Biol* **67**, 378-380 (2002).
11. Hughes, A.C. *et al.* Using echolocation calls to identify Thai bat species: Vespertilionidae, Emballonuridae, Nycteridae and Megadermatidae. *Acta Chiropt* **13**, 447-455 (2011).
12. Balete, D.S. Food and roosting habits of the lesser false vampire bat, *Megaderma spasma* (Chiroptera: Megadermatidae), in a Philippine lowland forest. *Asia Life Sci Suppl* **4**, 111-129 (2010).
13. Advani, R. Seasonal fluctuations in the feeding ecology of the Indian false vampire, *Megaderma lyra lyra* (Chiroptera: Megadermatidae) in Rajasthan. *Z Saugetierkd* **46**, 90-93 (1981).
14. Carter, G.G., Logsdon, R., Arnold, B.D., Menchaca, A. & Medellin, R.A. Adult vampire bats produce contact calls when isolated: acoustic variation by species, population, colony, and individual. *PLoS One* **7**, e38791 (2012).
15. Bobrowiec, P.E.D., Lemes, M.R. & Gribel, R. Prey preference of the common vampire bat (*Desmodus rotundus*, Chiroptera) using molecular analysis. *J Mammal* **96**, 54-63 (2015).
16. Carter, G.G., Coen, C.E., Stenzler, L.M. & Lovette, I.J. Avian host DNA isolated from the feces of white-winged vampire bats (*Diaemus youngi*). *Acta Chiropt* **8**, 255–274 (2006).
17. Gonzalez-Terrazas, T.P. *et al.* How nectar-feeding bats localize their food: Echolocation behavior of *Leptonycteris yerbabuenae* approaching cactus flowers. *PLoS One* **11**, e0163492 (2016).
18. Cole, F.R. & Wilson, D.E. *Leptonycteris yerbabuenae*. *Mamm Species* **797**, 1-7 (2006).
19. Kalko, E.K.V. Neotropical leaf-nosed bats (Phyllostomidae): 'whispering' bats or candidates for acoustic surveys? in *Bat Conservation International* (eds Brigham, R.M., Kalko, E.K.V., Jones, G., Parsons, S. & Limpens, H.J.G.A.) 63-69 (Austin, 2004).
20. Willig, M.R., Camilo, G.R. & Noble, S.J. Dietary overlap in frugivorous and insectivorous bats from edaphic cerrado habitats of Brazil. *J Mammal* **74**, 117-128 (1993).
21. Deshpande, K. & Kelkar, N. Acoustic identification of *Otomops wroughtoni* and other free-tailed bat species (Chiroptera: Molossidae) from India. *Acta Chiropt* **17**, 419-428 (2015).
22. Leelapaibul, W., Bumrungsri, S. & Pattanawiboon, A. Diet of wrinkle-lipped free-tailed bat (*Tadarida plicata* Buchannan, 1800) in central Thailand: insectivorous bats potentially act as biological pest control agents. *Acta Chiropt* **7**, 111-119 (2005).
23. Zhang, C. *et al.* Geographical variation in the echolocation calls of bent-winged bats, *Miniopterus fuliginosus*. *Zoology (Jena)* (2018).
24. Hiryu, S., Hagino, T., Fujioka, E., Riquimaroux, H. & Watanabe, Y. Adaptive echolocation sounds of insectivorous bats, *Pipistrellus abramus*, during foraging flights in the field. *J Acoust Soc Am* **124**, EL51-6 (2008).
25. Lee, Y. & Lee, L. Food habits of Japanese pipistrelles *Pipistrellus abramus* (Chiroptera: Vespertilionidae) in northern Taiwan. *Zool Stud* **44**, 95-101 (2005).
26. Zhao, H. *et al.* The evolution of color vision in nocturnal mammals. *Proc Natl Acad Sci U S A* **106**,

8980-5 (2009).
